# Targeting PLK1 as a novel chemopreventive approach to eradicate preneoplastic mucosal changes in the head and neck

**DOI:** 10.18632/oncotarget.17880

**Published:** 2017-05-16

**Authors:** D. Vicky de Boer, Sanne R. Martens-de Kemp, Marijke Buijze, Marijke Stigter-van Walsum, Elisabeth Bloemena, Ralf Dietrich, C. René Leemans, Victor W. van Beusechem, Boudewijn J.M. Braakhuis, Ruud H. Brakenhoff

**Affiliations:** ^1^ Department of Otolaryngology-Head and Neck Surgery, VU University Medical Center, Cancer Center Amsterdam, Amsterdam, The Netherlands; ^2^ Department of Pathology, VU University Medical Center, Cancer Center Amsterdam, Amsterdam, The Netherlands; ^3^ Department of Maxillofacial Surgery/Oral Pathology, Academic Center for Dentistry Amsterdam (ACTA), Amsterdam, The Netherlands; ^4^ German Fanconi-Anemia-Help e.V., Unna-Siddinghausen, Germany; ^5^ Department of Medical Oncology, VU University Medical Center, Cancer Center Amsterdam, Amsterdam, The Netherlands

**Keywords:** preneoplastic fields, head and neck squamous cell carcinoma, siRNA screening, targeted treatment, polo-like kinase 1

## Abstract

Head and neck squamous cell carcinomas (HNSCC) and local relapses thereof develop in preneoplastic fields in the mucosal linings of the upper aerodigestive tract. These fields are characterized by tumor-associated genetic changes, are frequently dysplastic and occasionally macroscopically visible. Currently, no adequate treatment options exist to prevent tumor development. Array-based screening with a panel of tumor-lethal small interfering RNAs (siRNAs) identified *Polo-like kinase 1* (*PLK1*) as essential for survival of preneoplastic cells. Inhibition of PLK1 caused cell death of preneoplastic and HNSCC cells, while primary cells were hardly affected. Both siRNAs and small molecule inhibitors caused a strong G2/M cell cycle arrest accompanied by formation of monopolar spindles. In a xenografted mouse model PLK1 caused a significant tumor growth delay and cures, while chemoradiation had no effect. Thus, PLK1 seems to be a promising target for chemopreventive treatment of preneoplastic cells, and could be applied to prevent HNSCC and local relapses.

## INTRODUCTION

Worldwide around 600,000 cases of head and neck squamous cell carcinoma (HNSCC) arise each year, forming about 5% of all cancer cases [1]. HNSCCs develop in the mucosal linings of the larynx, hypopharynx, oropharynx or oral cavity, and most important risk factors are tobacco smoking, excessive alcohol consumption as well as infection with high-risk human papillomavirus (HPV). Patients have a 5-year survival rate of 50-60% mainly due to the high incidence of local relapses and second primary tumors that are difficult to treat curatively [2].

An important phase in HNSCC development is the formation of genetically altered precancerous fields. These fields develop in several steps, starting when a mucosal stem cell undergoes mutations and other (epi)genetic alterations. These mutations are subsequently transferred to the daughter transit-amplifying and differentiating cells, forming a genetically altered clonal unit (‘patch’) in the mucosal linings. Through further accumulation of genetic changes these patches overgrow the normal mucosa and form fields of genetically altered cells that may reach dimensions of 10 cm in diameter. Despite the cancer-related genetic alterations in these cells, such as *TP53* mutations and losses of the chromosomal arms 3p, 9p and 17p, these preneoplastic fields are not yet invasive [2].

Preneoplastic fields are sometimes macroscopically visible as leukoplakia (white lesions) or erythroplakia (red lesions; [3]), however most fields are invisible to the naked eye [4, 5, 6]. Others demonstrated the presence of preneoplastic fields by using a device that detects autofluorescence. Changes in autofluorescence indicated neoplastic fields varying in size in 95% of the tested HNSCC patients [7]. The presence of these fields forms a high risk for local relapses and second primary tumors, especially since without visualization these imperceptible preneoplastic cells surrounding the tumor may stay behind when the primary tumor is resected [8, 9, 10, 11]. Intriguingly, resection of fields by guidance of autofluorescence had a significant impact on the local recurrence-free survival [12]. This preliminary observation stresses the urge of a suitable preventive treatment to eradicate preneoplastic fields when diagnosed by either clinical inspection, autofluorescence, histological examination or genetic analysis. As these fields are preneoplastic and not invasive cancer, preventive treatments should be effective but with limited toxicity. Radiotherapy is therefore not indicated. Surgical excision is an option, but in cases where visible lesions qualify for resection, they frequently recur. Targeted treatment approaches are less toxic than classical cytotoxic agents, and seem most promising in this respect. To develop these, druggable gene targets should be identified as a first step.

A prerequisite for the identification of promising target genes is the availability of preneoplastic cell models. Previously, a preneoplastic cell line, VU-preSCC-M3, was generated by our group [13]. This cell line was established from the resection margins of a 67 year-old patient with a T4aN0 tumor in the glottic larynx. The cells contained a nonsense mutation in *TP53*, loss of heterozygosity (LOH) of chromosome arms 3p and 9p and were shown to be preneoplastic in an organotypic cell culture model. Hence, this cell line model allows testing of novel treatment approaches. In recent years we developed more models and obtained these from other research groups as well [14].

The second requirement to develop an appropriate treatment is the presence of suitable target genes that can be exploited. The number of druggable driving oncogenes in HNSCC is disappointingly low and confined to *PIK3CA* mutations in less than 15% [15]. Hence, we relied on high-throughput functional genomics, a well-validated approach for the identification of essential genes [16, 17, 18]. Using this approach, we previously identified over 300 siRNAs that target genes essential for lung as well as head and neck cancer cells [17]. Here, we examined these ‘tumor-lethal’ siRNAs in preneoplastic cell cultures and identified genes that can be employed to potentially target preneoplastic cells. To be able to establish a clinical perspective, several available small molecule inhibitors were tested and the underlying working mechanism was investigated.

## RESULTS

### Array-based siRNA screening with a ‘tumor-lethal’ library reveals genes essential for preneoplastic cells

To identify possible targets for the treatment of preneoplastic fields we performed an array-based screen with 319 previously identified [17] ‘tumor-lethal’ siRNAs using the preneoplastic cell line VU-preSCC-M3 [13] (Supplementary Figure 1). These siRNAs were ordered as a sublibrary and applied array-based in separate wells as pools of four siRNAs targeting the same gene. The originally screened tumor cell lines VU-SCC-120 and SW1573 were analyzed in parallel as reference. To identify the genes that are essential for cell survival we set the cut-off at ≥50% decrease in cell viability relative to the window of the positive (e.g. si*UBB*) and negative controls (siCONTROL). In case of SW1573, a non-small cell lung cancer (NSCLC) cell line, a total of 175 out of 319 siRNAs (55%) met this criterion, for VU-SCC-120 (HNSCC) we identified 211 lethal siRNAs (66%) (Table 1 and Supplementary Table 1). Combined, 245 out of 319 siRNAs (77%) showed a lethal effect in either the HNSCC and/or the NSCLC cell line with the used, relatively strict, criterion.

**Table 1:** Re-screen of 319 ‘tumor-lethal’ siRNAs revealed 98 genes to be essential for the preneoplastic cell line VU-preSCC-M3

Original genome-wide screen^a^								
	SW1573 only^a^197 essential siRNAs		Overlap^a^62 essential siRNAs		VU-SCC-120 only^a^60 essential siRNAs		Total^a^319 essential siRNAs	
	Hits (n)	Percentage	Hits (n)	Percentage	Hits (n)	Percentage	Hits (n)	Percentage
**Rescreenb SW1573**	104	53%	54	87%	17	28%	175	55%
**Rescreenb VU-SCC-120**	101	51%	59	95%	51	85%	211	66%
**Rescreenb VU-preSCC-M3**	43	22%	43	69%	12	20%	98	31%

^a^Hits identified in original genome-wide screens of two tumor cell lines; originating from SW1573 or VU-SCC-120 alone or as overlapping hits of both cell lines [17]. Of note, in the previous study, siRNA hits were identified by robust Z-score analysis, while in this validation analysis a more stringent cut-off of >50% cell death was considered, which will impact the true validation percentages.

^b^Re-screening of 319 ‘tumor-lethal’ siRNAs in two tumor cell lines and one preneoplastic cell line.

Hits accumulated from the originally genome-wide screened tumor cell lines VU-SCC-120 and SW1573 were retested in the original tumor cells and the precancerous cell line VU-preSCC-M3. In total, 98 siRNAs (31%) have a lethal phenotype in the analyzed preneoplastic cell line.

**Figure 1:**
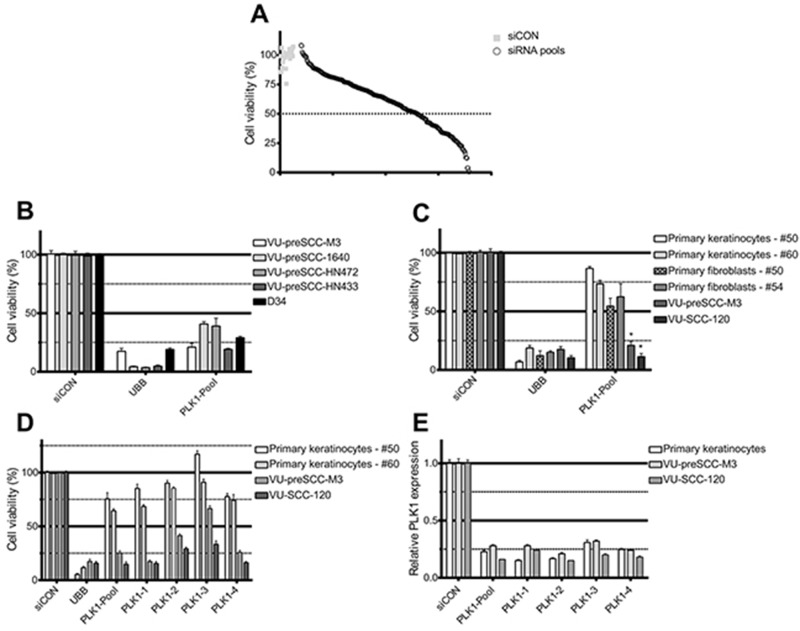
siRNA screening reveals *PLK1* as a selective therapeutic target in preneoplastic and HNSCC cells **(A)** Plot of the effect on cell viability of the 319 lethal siRNA SMARTpools and controls screened in VU-preSCC-M3. Black open dots represent the median values of the 319 pools tested in triplicate, and grey squares all siCONs (negative controls). The dotted line indicates 50% survival, and 98 siRNA pools were below this value. **(B)** In addition to VU-preSCC-M3, four other preneoplastic cell lines also demonstrated ≥50% decrease of cell viability after transfection with the PLK1 SMARTpool. **(C)** Primary cell cultures of non-tumor tissue samples were less sensitive to knockdown of *PLK1*. VU-preSCC-M3 and VU-SCC-120 were included as reference. The differences between fibroblasts and keratinocytes versus VU-preSCC-M3 and VU-SCC-120 cells were highly significant. ^*^ *P*<0.003; Mann–Whitney-U-test. **(D)** Deconvolution of the SMARTpool in the four separate siRNAs showed an almost identical phenotype for three out of four separate siRNAs for all cell cultures tested. **(E)** The SMARTpool and the four individual siRNAs directed against *PLK1* resulted in at least 70% knockdown of *PLK1* mRNA levels. Bars represent the mean values of three independent experiments typically executed in triplicate in B, C and D. The error bars represent the SEM. Bars in figure E represent mean values of two independent measurements, with error bars representing the SD.

For the preneoplastic cell line, VU-preSCC-M3, we applied the same criterion for lethal hit identification, and identified 98 lethal siRNAs (31%) that target apparently genes essential for preneoplastic cells (Figure 1A, Table 1 and Supplementary Table 1). Of these 98 hits, 43 (44%) originated from the SW1573 genome-wide screen, 12 (12%) from the VU-SCC-120 based screen, and 43 (44%) from overlapping hits of the two originally screened cell lines (Table 1). Within the set of 98 essential genes that showed a lethal phenotype in the preneoplastic cells, several functional clusters could be identified, including a cluster of genes regulating the mitotic phase of the cell cycle (Supplementary Figure 2; STRING database version 10).

### PLK1 seems a potential target for HNSCC treatment and prevention

Next, a further selection of promising target genes was made, based on the existence of small molecule inhibitors using available databases, and the lethal effect of the siRNAs. One of the siRNAs with the strongest effect targets *Polo-like kinase 1* (*PLK1*). We explored the future outlook of PLK1 inhibition as therapeutic strategy for preneoplastic fields (Supplementary Figure 1). *PLK1* encodes a protein with multiple functions in the regulation of the cell cycle and is a known target gene for cancer treatment, and druggable with several small molecule inhibitors currently tested in clinical trials. PLK1 is overexpressed in a variety of tumor types [19] and a number of studies indicate a correlation between the level of *PLK1* expression in tumors and prognosis [20, 21, 22]. Indeed, we detected elevated levels of *PLK1* expression in HNSCC tumors by analyzing microarray mRNA profiles of a panel of 22 HNSCC tumors with matched normal mucosa samples. We found higher expression of *PLK1* in all tumor samples, with on average a 2.26-fold increase compared to normal mucosa (*P*<0.0001 Student *t* test; Supplementary Figure 3). In contrast, the RNA expression levels of the two most closely related PLK family members, *PLK2* and *PLK3*, were both lower in the tumor specimens (0.66 and 0.48-fold, respectively). *PLK4* is also expressed at a higher level in tumors, although with a lower fold increase than *PLK1* (1.44-fold). Of note, only *PLK1* was identified as a hit in the initial screens, and none of the other members of the family of polo-like kinases (Plks). The expression data confirms that PLK1 inhibition could indeed serve as a therapeutic strategy for HNSCC treatment in patients.

### Cell death induced by *PLK1* knockdown is cancer- and precancer-specific

To assess the more general suitability of PLK1 inhibition for treatment of preneoplastic fields we tested in addition to VU-preSCC-M3 other preneoplastic cell models, generated by us or by others [14] (Supplementary Table 2). RNAi-mediated *PLK1* knockdown had the same effect on cell viability in these models, with a 60% to 80% decrease in viable cells compared to the negative control (Figure 1B).

Next, we defined the possible toxic effects of PLK1 inhibition, at least *in vitro*, by *PLK1* knockdown on non-malignant cells. Only minor effects on cellular viability were observed after *PLK1* knockdown in both primary mucosal keratinocytes and fibroblasts of several healthy donors (Figure 1C). Of note, PLK1 protein expression was relatively low in primary keratinocytes (Supplementary Figure 4A), while the doubling time was comparable, at least in the first passages (Supplementary Table 2). Deconvolution of the PLK1 SMARTpool to four distinct siRNAs targeting different sequences of the mRNA, showed significant and generally similar effects on cell viability. Only siRNA 3 was less effective (Figure 1D). As 3 out of 4 distinct siRNAs showed a >50% decrease of cell viability for VU-preSCC-M3, *PLK1* was considered a bona fide target. Knockdown analysis by RT-qPCR demonstrated a decrease in mRNA expression of approximately 70-80% (Figure 1E). In addition, the protein levels also decreased by more than 70% (Supplementary Figure 4B).

### Potency of small molecule PLK1 inhibitors *in vitro*

**Figure 2:**
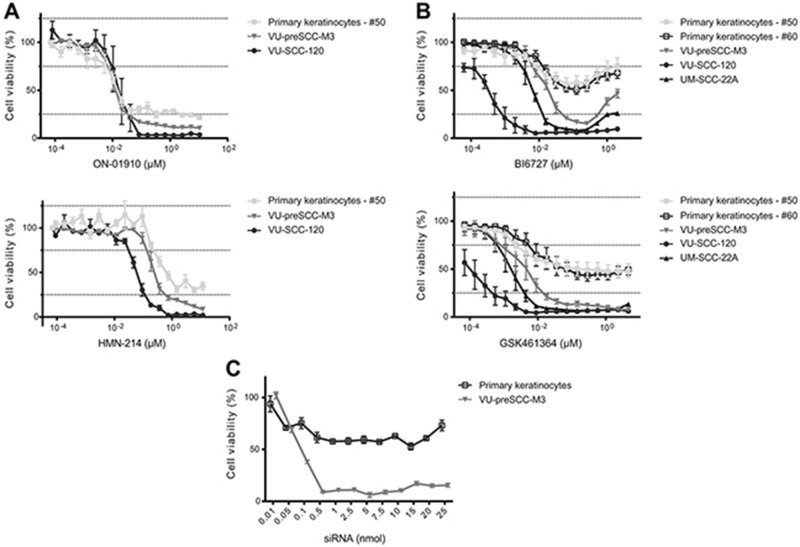
Selective PLK1 inhibitors show *in vitro* a therapeutic window between (pre)cancerous cells and primary cells **(A)** Treatment of cell cultures with ON-01910 (top) and HMN-214 (bottom) resulted in similar responses of primary, preneoplastic and tumor cells, with only a minor therapeutic window. **(B)** Treatment with two other small-molecule inhibitors, BI6727 (top) and GSK461364 (bottom) showed a large difference between primary cells versus preneoplastic and tumor cells. **(C)** As a reference of the effect of PLK1 inhibition by small molecule inhibitors we analyzed a serial dilution range of the PLK1 SMARTpool siRNAs from 0.01 till 25 nmol. The siCON values were set at 100%, whereas the UBB control showed more than 80% reduction of cell viability (not shown). All small molecule graphs (in panels A and B) represent mean values of three independent experiments performed with triplicates per experiment, and error bars indicate the SEM. Panel C represents mean values of three independent measurements, with error bars representing the SEM.

Several small molecule inhibitors of PLK1 are being evaluated in clinical trials and we selected four of these compounds to test on tumor (VU-SCC-120 and UM-SCC-22A) and preneoplastic (VU-preSCC-M3) cell lines, as well as on primary keratinocytes. Two of these drugs, i.e. BI6727 (Volasertib) and GSK461364, are potent competitive inhibitors of ATP-binding, ON-01910 (Rigosertib) is a non-competitive ATP inhibitor and HMN-214 is believed to interfere with the spatial distribution of PLK1 [23]. Strikingly, the *in vitro* cell survival profiles after treatment with these four small molecule inhibitors differed markedly, resulting in two types of responses. ON-01910 and HMN-214 strongly decreased viability of tumor, preneoplastic and primary healthy cells (Figure 2A). In contrast, BI6727 and GSK461364 treatments were less toxic to primary keratinocytes compared to tumor and preneoplastic cells (Figure 2B). Since not all inhibitors showed the same effect, with respect to profile and the window between preneoplastic and normal keratinocytes, a dilution range of the PLK1 siRNA SMARTpool was made to compare the small molecule and siRNA dose response curves (Figure 2C). The dose response curves of BI6727 and particularly GSK461364 showed the closest resemblance to the siRNA profile, which suggests the highest target specificity.

### G2/M phase arrest and improper spindle formation after PLK1 inhibition

**Figure 3:**
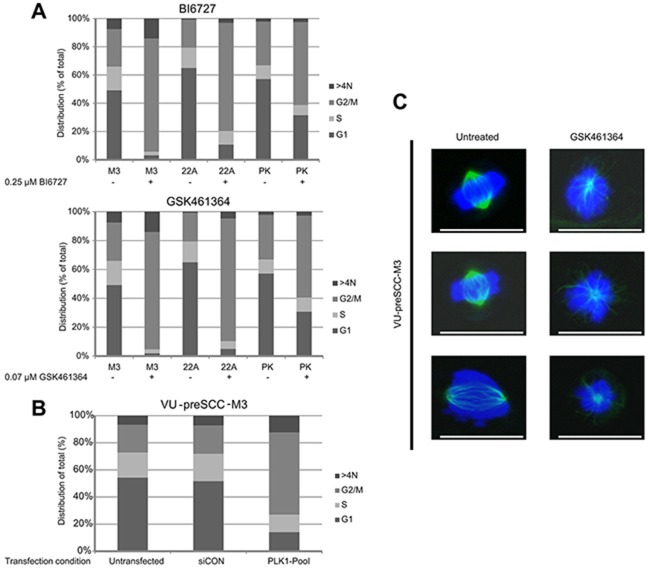
PLK1 inhibition induces a G2/M arrest in (pre)cancerous cells, together with loss of bipolar spindle formation **(A)** VU-preSCC-M3 and UM-SCC-22A as a reference, were exposed to BI6727 and GSK461364. This resulted in a strong G2/M arrest for both cell lines. Primary keratinocytes (PK) showed a less pronounced G2/M arrest after drug treatment. Bars represent the percentage of cells in a certain phase as indicated. Mean percentages of three independent experiments are shown. Data was analyzed with the Poisson equation and demonstrated a significant difference between the cell lines and the primary cells, for both GSK461364 and BI6727 (*P*<0.001). **(B)** Similar as to the inhibitors, VU-preSCC-M3 cells transfected with the siRNA SMARTpool directed against PLK1 undergo a G2/M arrest. Bars represent the percentages of cells similar as indicated in A. **(C)** VU-preSCC-M3 cells were treated with 0.07 μM GSK461364 and visualization of α-tubulin showed disorganized monopolar spindles in almost all cells. In contrast, untreated cells displayed only normal bipolar arranged spindles. Representative pictures of untreated and treated cells are shown, scale bars indicate 25 μm.

To explore the cellular effects of PLK1 inhibition on HNSCC and precancer mucosal cells, UM-SCC-22A and VU-preSCC-M3 were exposed to BI6727 (0.25 μM) or GSK461364 (0.07 μM) and after 24 hours cell cycle profiles were analyzed using flow cytometry. In both cases a G2/M arrest was seen in approximately 90% of the tumor as well as the preneoplastic cells. In addition, we noted a small increase in polyploidy, cells with a DNA content >4N (Figure 3A and Supplementary Figure 5). In contrast to the tumor and preneoplastic cells, primary mucosal keratinocytes showed only a modest G2/M arrest when treated with the same concentrations of BI6727 and GSK461364 (Figure 3A). This is in line with their insensitivity to siRNA mediated knockdown. These results suggest that primary cells have mechanisms to rescue proper cell division with reduced activity of PLK1 and thereby circumvent the G2/M arrest, a characteristic that apparently has been lost during carcinogenesis.

To confirm the direct involvement of PLK1 in the observed cell cycle arrest, we also tested cell cycle distribution patterns after RNAi-mediated knockdown. Flow cytometry analysis of VU-preSCC-M3 after transfection with the PLK1 SMARTpool resulted in an arrest in G2/M comparable to that of the small molecule inhibitors (Figure 3B).

Next, we tested whether PLK1 inhibition in preneoplastic cells affects spindle formation in a similar way as was previously demonstrated for tumor cells [24, 25]. Dividing untreated preneoplastic cells all showed normal spindle assembly, monopolar spindles were observed in 0 out of 67 counted cells (0%). Most cells treated with GSK461364 did no longer assemble proper spindles, monopolar spindles were observed in 85 of 92 counted cells (92%) in the presence of the inhibitor (*P*<0.0001; Fisher Exact test; Figure 3C). These results indicate improper spindle formation in VU-preSCC-M3 cells by PLK1 inhibition, identical as observed in tumor cells.

### Preclinical assessment of PLK1 inhibitors demonstrates efficacy in a HNSCC animal model

**Figure 4:**
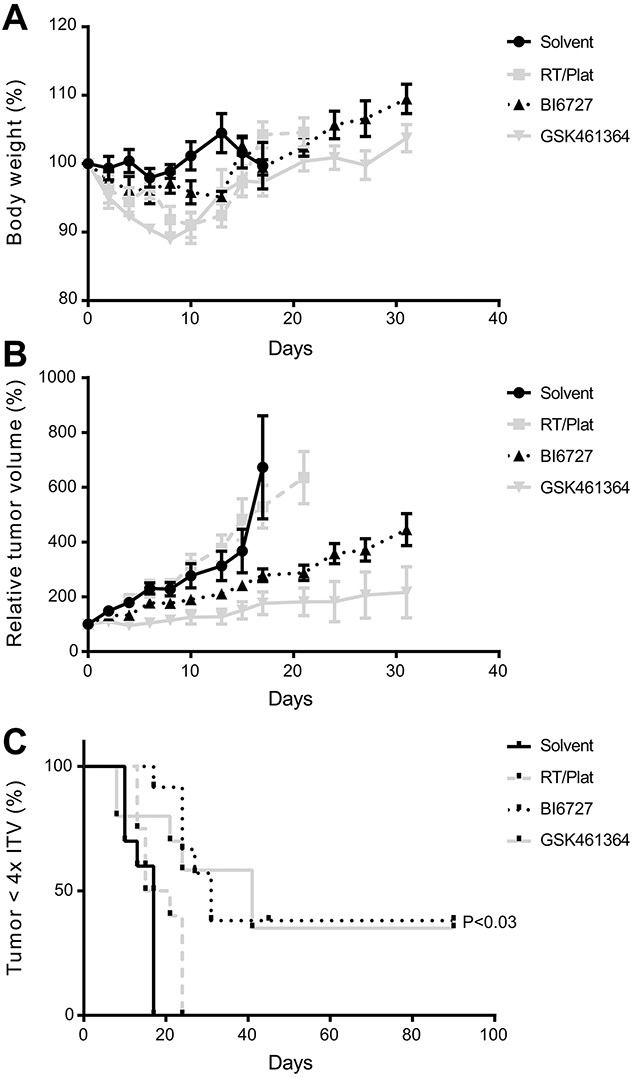
Efficacy of PLK1 inhibitors in an HNSCC xenograft model **(A)** Relative changes in body weight showed tolerable weight losses after treatment. After treatment, mice recovered quickly to their initial weight and gained additional weight. **(B)** Significant suppression of tumor growth results from the treatment with either GSK461364 or BI6727. In case of GSK461364 two complete responses were observed, where BI6727 showed one mouse with a likely cure at both sites. Both inhibitors were significantly more effective than the solvent group as well as the group treated with chemoradiation (*P*<0.006; Student *t* test). Both graphs (A and B) represent mean values per group (n=6) and error bars indicate the SEM. **(C)** Survival curves, based on the time until an individual tumor reaches 4x the initial tumor volume (4xITV). Both GSK461364 and BI6727 showed a significant improvement of survival (P=0.025 and 0.0002, respectively; Log-rank test).

As described above, elevated expression levels of PLK1 in patient material supports the potential of PLK1 as therapeutic target. To explore this in a more clinical relevant setting, we used a HNSCC mouse model treated with the two most promising inhibitors according to our *in vitro* data, BI6727 and GSK461364. First we determined the maximum tolerable dose (MTD; data not shown). Next, mice bearing UM-SCC-22A xenograft tumors were treated with the two selected inhibitors (Supplementary Table 3) based on the pilot MTD data we obtained, and compared to solvent and chemoradiation protocols. In general, treatment was tolerated well for PLK1 inhibitors as judged by clinical signs and body weight changes (Figure 4A). One mouse in the GSK461364 treated group showed more weight loss and had to be discontinued from treatment and was euthanized according humane endpoint criteria (day 8), but was kept in the data analysis.

Treatment resulted in both groups in a significant reduction of tumor volume, when compared to the control group and also to the group treated with radiotherapy and cisplatin (*P*<0.006 at day 17; Student *t* test; Figure 4B). Survival, based on the time to reach 4 times the initial tumor volume (4xITV), significantly increased (*P*<0.03; Log-rank test) for both groups treated with a small molecule inhibitor compared to solvent, while chemoradiation did not show beneficial effects (Figure 4C). In the BI6727 group we observed one likely cure, while in the GSK461364 treated group two mice showed a complete response, providing cures in 2 of 6 treated mice.

## DISCUSSION

Cancer in the head and neck may be preceded by visible or macroscopically invisible lesions, and as indicated in previous studies these preneoplastic lesions are an important source for primary tumors in the general population and relapses in treated HNSCC patients [8, 9, 10, 11]. An effective treatment of these fields would be of great benefit, but as result of their preneoplastic nature only treatments with a low toxicity profile are acceptable. Unfortunately, there are no suitable therapeutic strategies to eradicate these fields. Visible lesions can be treated by surgery or laser, but efficacy of these modalities is questionable [26], as the lesions are larger than is visible and frequently recur. Very recently fluorescence guided resections showed promising results and provided the proof of concept that adequate treatment of fields will reduce tumor burden [12]. To identify therapeutic targets for these precancerous fields, we used array-based screening of previously identified siRNAs that are lethal in tumor cell lines, and identified 98 siRNAs that eradicate preneoplastic cells in culture. For comparison we also re-tested the two original tumor cell lines that were previously screened, and many of the hits could be confirmed. For both cell lines more than half of the tested siRNAs showed a significant effect with the relatively strong criterion of >50% cell death. In general, the preneoplastic cells seem to be less sensitive to knockdown with these ‘tumor-lethal’ siRNAs, since only 31% of the siRNAs gave rise to significant cytotoxicity in these cells. This suggests that the larger mutational burden in tumor cells makes them more vulnerable for knockdown of specific genes, an intriguing observation. Generally preneoplastic cultures show less genetic changes than tumor cell lines (manuscript in preparation).

In this study, *PLK1* was selected as promising target gene, since its suppression resulted in profound growth inhibiting effects in both tumor and preneoplastic cells but much less in primary cells. The fact that PLK1 is found to be overexpressed in HNSCC specimen and that it also seems to be overexpressed in neoplastic cell models *in vitro*, adds up to the concept of PLK1 as a promising and selective target. In the past, others demonstrated these elevated levels as well and even showed that a high PLK1 expression level is associated with a poor prognosis and a lower overall-survival time [27]. The protein entered the spotlight as target molecule for several tumor types, and our data support the important role of PLK1 in HNSCC cells as well. Here, we show as well that it may serve as therapeutic target in preneoplastic cells likewise, a totally novel concept.

PLK proteins are important regulators of the cell cycle and cell division, and PLK1 in particular plays critical roles. Both the activation of PLK1 by posttranslational modifications, its cellular localization, and the overall expression levels determine the precise role during the different phases of the cell cycle, including mitotic entry, centrosome maturation and bipolar spindle formation [19, 28]. The other Plk family members seem to have a less important role, as indicated by their expression profiles in HNSCC tumors. Moreover, in the original genome-wide screen performed on VU-SCC-120 only siRNAs silencing *PLK1* gave rise to a lethal phenotype, which was not observed for siRNAs targeting *PLK2*, *3* or *4* [17].

In a clinical setting, inhibition of PLK1 might be useful for both HNSCC tumor treatment as well as prevention of tumors and local relapses [5, 9]. In particular, patients who are at high risk for developing head and neck cancer and local relapses could be enrolled in future trials. Risk assessment can be based on the presence of genetic alterations in visible lesions or the surgical margins, for instance losses of chromosome arms 3p and 9p [8]. Our data suggests that treatment of visible leukoplakia lesions might be successful as well. With an annual transformation rate of 1-2% per year, the presence of visible leukoplakia lesions does not directly lead to an immediate cancer risk [3, 29], but after longer follow-up 50% of these patients may develop cancer. To use PLK1 inhibition in a preventive treatment setting, only patients with precancerous changes at high risk of malignant transformation, which contain genetic changes at multiple chromosomal arms or aneuploidy, are eligible [30, 31].

When we applied siRNA-mediated *PLK1* knockdown, differences in response were observed between tumor and premalignant cells versus primary cells. However, when we analyzed the selected panel of small molecule inhibitors, their profiles did not always mimic that of siRNA knockdown, which we consider as the most specific way of target inhibition. Only the inhibitors BI6727 and GSK461364 showed profiles *in vitro* that were comparable to the siRNAs, evidence of target selectivity. This selectivity was confirmed by the specific effects on the cell cycle distribution of primary cells treated with GSK461364 or BI6727. The G2/M arrest of keratinocytes after drug treatment was significantly smaller than that demonstrated for the preneoplastic cells and HNSCC tumor cells. These differences are not depending on the proliferation rates of the cells, as the primary cells have a comparable doubling time as the VU-preSCC-M3 cells (Supplementary Table 2). Likely, it relates to the abrogation of G1/S cell cycle control by loss of p16 and the absence of functional p53, since VU-preSCC-M3 contains a nonsense *TP53* mutation [13].

It has been shown by others that PLK1 inhibition in primary cells causes senescence via a p53 dependent pathway, which might apply to the primary cells used in our studies as well. This can be missed when cell viability is examined by metabolic assays as senescent cells remain metabolically active [32, 33]. However, senescence seems not to have any clinical consequences. Phase I studies of BI6727 and GSK461364 in patients with solid tumors demonstrated moderate antitumor effects, while dose limiting toxicities were mainly myelosuppression, and not mucositis which is a clinical sign that could result from senescence [34, 35]. Since, there are no established precancer mouse models, we used a HNSCC animal model to demonstrate the clinical applicability, which was clearly demonstrated. Both inhibitors showed a significant delay of tumor growth, and even 2 cures of 6 mice for GSK461364 and 1 cured mouse for BI6727. These two inhibitors are interesting options for chemopreventive treatment of high-risk preneoplastic fields, but also for HNSCC patients. Given the toxicity profile of these inhibitors, selection of patients at high risk for malignant transformation seems required.

There is one potential undesirable aspect of BI6727 that requires clinical evaluation: at higher doses cell viability increased *in vitro* (Figure 2B). Examination of cell cycle distribution in the presence of these higher concentrations (>2μM) revealed that the arrest in the G2/M phase also diminished, suggesting that less cells suffer from PLK1 inhibition (data not shown). We assume that this is due to off-target effects on kinases that interfere with the effects of PLK1 inhibition, although it was shown previously that 10 μM BI6727 did not have effects on a panel of 63 kinases [36]. Nonetheless, multiple other candidate kinases have not been tested and some may be active in the molecular mechanisms that lead to cell death after PLK1 inhibition.

In conclusion, PLK1 is a promising drug target for chemopreventive trials to eradicate high risk preneoplastic fields, and to prevent tumors in leukoplakia patients and local relapses in treated HNSCC patients. To our knowledge we are the first to report on a novel targeted chemoprevention approach for mucosal preneoplastic changes based on a rational design.

## MATERIALS AND METHODS

Collection and culture of patient material was approved by the Institutional Review Board of the VU University Medical Center (protocol 2008-71). Tissue samples of patients with HNSCC were collected after informed consent. Use of residual tissue of surgical non-tumor specimens was carried out according to the guidelines for analyzing human samples of the Dutch Medical Scientific Societies (www.federa.org).

### Cell lines

Primary cells were collected from excised uvulas of healthy adults as previously described [13]. Primary fibroblasts were cultured in Dulbecco's Modified Eagle Medium (DMEM, Lonza), with 10% fetal calf serum (Lonza) and 2 mmol/L L-glutamine (Lonza). The primary oropharyngeal keratinocytes were cultured in serum-free KGM medium (KGM-SFM, Gibco), supplemented with 0.1% BSA (MP biomedicals), 25 mg bovine pituitary extract (Gibco), 2.5 μg human recombinant EGF (Gibco), 250 μg Amphotericin B (Gibco) and 250 μg Gentamycin (Sigma-Aldrich). VU-preSCC-M3 was previously generated [13]. D34, a preneoplastic cell line derived from a leukoplakia lesion, was a generous gift of Dr. K. Hunter in 2014 (University of Sheffield, Sheffield, UK) and was previously described [14]. The other preneoplastic cell lines, VU-preSCC-1640, VU-preSCC-HN433 and VU-preSCC-HN472 were generated and characterized at our own lab (Supplementary Table 2) as described previously [13]. Detailed molecular characteristics of these and other preneoplastic cultures will be published elsewhere (manuscript in preparation). All preneoplastic cell lines were cultured in KGM-SFM. VU-SCC-120, previously described as 93VU120 was established by Hermsen *et al*. [37]. UM-SCC-22A was obtained from Dr. T. Carey in 1986 (University of Michigan, Ann Arbor, MI [38]). SW1573 was obtained from the American Type Culture Collection in 2009. The tumor cell lines were cultured in DMEM, complemented with 5% fetal calf serum and 2 mmol/L L-glutamine. All cells were cultured in a humidified atmosphere at 37°C under 5% CO_2_, and used within three months after thawing. Authentication of the cell lines was conducted regularly based on morphological characteristics, *TP53* mutations and other genetic markers.

### siRNA re-screen

siRNAs were selected from previous genome-wide screens and subsequently tested in a sublibrary format on VU-preSCC-M3 and the two original cell lines used, VU-SCC-120 and SW1573 [17]. The sublibrary was ordered as siRNA SMARTpools derived from the siARRAY Human Genome library (Dharmacon, GE). From the original 362 siRNA pools, that were scored as hit based on Z-scores, 319 remained as bona fide genes and 43 were no longer in the product list as the genes were retired. All three cell lines were forward transfected in triplicate with 25 nmol of every SMARTpool, including individual positive and negative controls together with DharmaFECT1 (Dharmacon, GE). For VU-preSCC-M3, VU-SCC-120 and SW1573 0.1 μl, 0.03 μl and 0.02 μl DharmaFECT1 per well, respectively, was determined as being optimal. After 96 hours of incubation, cell viability was determined using the CellTiter-Blue (CTB) reagent assay (Promega) at a 1:1 dilution with medium. The reaction was stopped after 2 hours by adding 3% sodium dodecyl sulfate (Applichem Panreac). Fluorescence was measured with an Infinite F200 microplate reader (Tecan), with excitation wavelength set at 540 nm and emission wavelength at 590 nm.

### Screen data analysis

To identify siRNAs that caused significant loss of cell viability, raw median viability values were transformed into percentages for every cell line as follows. To correct for differences in transfection efficiency between cell lines the maximum achievable range of the positive controls compared to the negative controls was set at 100%. Wells with 50% or more reduction of cell viability were considered to represent valid hits. Next, we defined which of the remaining genes are druggable, making use of publicly available databases, and cherry-picking was performed based on the availability of small molecule inhibitors.

### Deconvolution and serial dilution analysis

Multiple cell lines were forward transfected with the target siRNAs and with the non-targeting siCONTROL#2 (siCON, Dharmacon, GE) and a SMARTpool directed against *Ubiquitin B (UBB*, Dharmacon, GE) [39], as negative and positive control, respectively. For deconvolution a PLK1 SMARTpool was used together with four separate siRNAs. At 96 hours post-transfection cell viability was measured as described above. Percentages of cell viability were calculated relative to the negative controls. For slower dividing cell lines such as VU-preSCC-HN433 and VU-preSCC-HN472, the incubation period after transfection was adjusted according to the doubling time of the cells to ensure at least three population doublings (Supplementary Table 2). The culture medium was refreshed during these longer incubations, to prevent starvation. For the siRNA dilution range, the target SMARTpool was diluted with 1x siRNA buffer (Dharmacon, GE) to obtain concentrations ranging from 0.01 nmol till 25 nmol.

### Expression microarray

Clinical specimens of a panel of 22 HNSCCs and corresponding macroscopically normal mucosa, adjacent to the tumor, were collected as described [40]. In short, 13 males and 9 females were included with an average age of 59.6 ±10.2 years (range 33-82). Specimens were obtained from the oral cavity (17 of 22, 77%) and the oropharynx (5 of 22, 23%) and all mucosa samples were judged free from dysplasia. All oropharyngeal tumors were HPV negative. RNA was isolated with TRIzol, analyzed on an Agilent 2100 Bioanalyzer (Agilent) and complementary DNA (cDNA) synthesis was performed (Agilent). Expression profiles were determined by microarray (4×44K Whole Human Genome Arrays G4112F) hybridization and readout was performed on a G2505B microarray scanner system (Agilent). Data analysis was performed as described previously [41]. Data have been uploaded to GEO with accession number GSE83519.

### Quantitative RT-PCR

RNA was collected 24 hours after transfection, with the RNeasy micro kit (Qiagen). cDNA was produced from 100 ng RNA with a cDNA reverse transcriptase kit (Applied Biosystems). For quantitative measurements of *PLK1* a 2x Universal PCR Mastermix (Applied biosystems) together with a gene-specific expression assay (Hs00153444_m1) was used. β-Glucuronidase (*GUSB*; Hs00939627_m1; [42]) was used as an internal reference. Reactions were performed on the ABI/Prism 7500 sequence detector system (Taqman-PCR, Applied Biosystems). Expression of *PLK1* was calculated relative to *GUSB* (ΔCt method).

### Western blot

Cells were lysed in RIPA buffer (Thermo Scientific) supplemented with a HALT protease and phosphatase inhibitor cocktail (Thermo Scientific) 24 hours after transfection with siRNAs. Lysates were loaded onto 4-20% Mini-Protean TGX gels (BioRad) and transferred to a PVDF membrane (Merck Millipore). Western blot was performed with rabbit anti-PLK1 (1/500, Cell Signaling) and mouse anti-β-actin (clone AC-15, 1/20,000, Sigma-Aldrich) antibodies. Proteins were visualized with the fluorescently labeled antibodies goat-anti-rabbit IRDye 800CW and goat-anti-mouse IRDye 680RD (1/5,000, LI-COR Biosciences). Blots were scanned on the Odyssey infrared imaging system (LI-COR Biosciences). Protein levels were standardized to the β-actin levels and quantification was performed with ImageJ Software (NIH).

### Dose response curves

Cells were treated with four small molecule inhibitors directed against PLK1, GSK461364 (Axon Medchem), BI6727 (Axon Medchem), ON-01910 (Selleckchem) and HMN-214 (Selleckchem), all dissolved in DMSO. Cells were treated with concentrations ranging from 0 till 12 μM, where concentrations of DMSO were always below 0.1%. After 72 hours of incubation the effects on cell viability were measured as described above.

### Cell-cycle analysis

Cells were treated for 24 hours with either 0.25 μM BI6727 or 0.07 μM GSK461364, or with 25 nmol of the siRNA SMARTpool or siCON. Next, cells were fixed with 70% ice-cold EtOH in PBS (Lonza). Before staining, cells were incubated with 50 μl RNAse (100 μg/ml, Sigma-Aldrich) for 30 minutes at room temperature. Then 200 μl Propidium Iodide (PI, 50 μg/ml, Sigma-Aldrich) was added and DNA content measurements were performed on a FACS BD LSR II Fortessa (BD Biosciences). Cell cycle distribution was analyzed with BD FACS DIVA software (V8.0.1) (BD Biosciences).

The data was analyzed using a generalized linear model with a Poisson error and a logarithmic link. The linear prediction included cell line and treatment effects, as well as an interaction between these two.

### Immunofluorescence staining of mitotic spindles

VU-preSCC-M3 cells were grown on 8-well Lab-Tek chamber slides (Thermo Fisher Scientific) and treated with 0.07 μM GSK461364 for 24 hours. The cells were fixed and stained to visualize the mitotic spindles as described [17]. Representative pictures were selected and background staining was filtered using ImageJ.

### HNSCC xenograft study

HNSCC cell line UM-SCC-22A was subcutaneously injected in both flanks of 8-week-old female athymic Nude-Foxn1^nu^ mice (Envigo), with 2 × 10^6^ cells per site. Approximately 4 weeks later, mice (n=6) were randomized into a solvent (control), BI6727, GSK461364 and a chemoradiation group (RT/Plat). The latter concomitantly treated with radiotherapy (2 Gy) and cisplatin (3 mg/kg), a frequently used treatment strategy in clinical care albeit with a much higher total irradiation dose. Both inhibitors were formulated in 10% DMSO and 10% solutol, which was used as the solvent given to the control mice. Mice were treated according to the scheme depicted in Supplementary Table 3. Tumors were measured every other day by calipers, and tumor volume was quantified with the formula (length x width x depth) x 0.5. Animal weight was determined as indicator of treatment toxicity. All animal experiments were performed according to Dutch and EU legislations, and the protocol (14-01) was approved by the Institutional Review Board on animal experimentation.

### Statistical analysis

Differences between groups were assessed by 2-tailed Student *t* test or Mann–Whitney-U test. Differences in treatment effect on cell cycle distribution were assessed by a generalized linear model with a Poisson error and a logarithmic link. Differences in spindle assembly were assessed by the Fisher exact test. Analysis of survival of the treated mice was performed by the Kaplan-Meier method, and differences between the groups was assessed by the Log-rank test (Mantel-Cox). A *P* value less than 0.05 was considered significant.

## SUPPLEMENTARY MATERIALS FIGURES AND TABLES





## Abbreviations

cDNA: complementary DNA
CTB: CellTiter-Blue
DMEM: Dulbecco's Modified Eagle Medium
GUSB: β-Glucuronidase
HNSCC: head and neck squamous cell carcinoma
HPV: human papillomavirus
ITV: initial tumor volume
LOH: loss of heterozygosity
MTD: maximum tolerable dose
NSCLC: non-small cell lung cancer
PI: Propidium Iodide
PK: Primary keratinocytes
PLK1: Polo-like kinase 1
Plks: polo-like kinases
RT/Plat: chemoradiation
siCON: siCONTROL
siRNAs: small interfering RNAs
UBB: Ubiquitin B
