# Supplementary material for: Targeting PLK1 as a novel chemopreventive approach to eradicate preneoplastic mucosal changes in the head and neck
Source: Oncotarget. 2017 May 16;8(58):97928–40. doi: 10.18632/oncotarget.17880 (PMC5716703; doi:10.18632/oncotarget.17880)
Supplement: Supplementary file 1 [file oncotarget-08-97928-s001.pdf]

Targeting PLK1 as a novel chemopreventive approach to eradicate preneoplastic mucosal changes in the head and neck

SUPPLEMENTARY MATERIALS

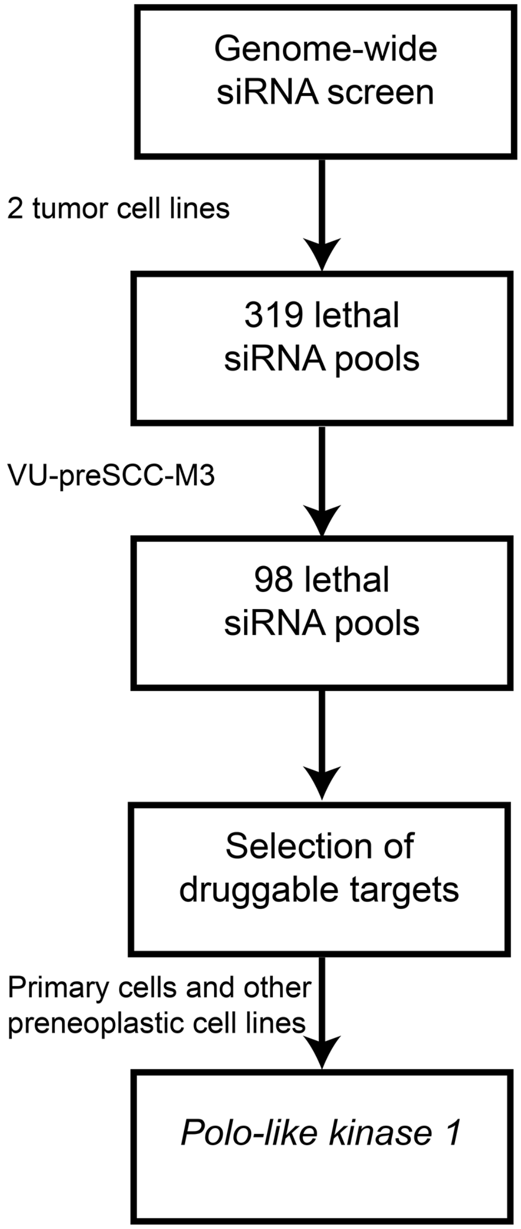

Supplementary Figure 1: Screening of VU-preSCC-M3 with a panel of 319 'tumor-lethal' siRNAs. Outline of the siRNA screening and candidate target identification procedure.

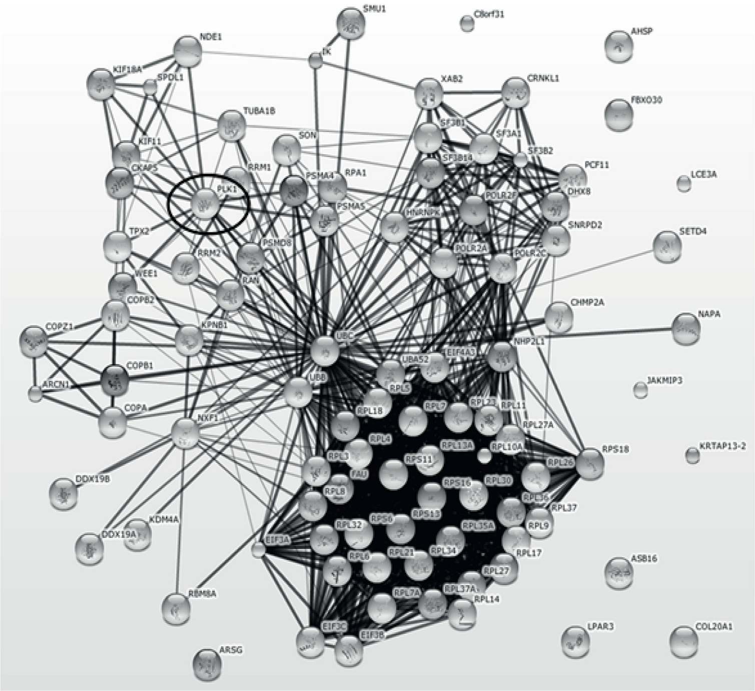

Supplementary Figure 2: Cluster analysis based on the 98 essential genes of preneoplastic cell line VU-preSCC-M3. Multiple pathways and cellular functions were found to be significantly overrepresented in this gene set, which includes mitosis, cell cycle regulation and ribosomal processing. *Polo-like kinase 1*, an important cell cycle regulator and a druggable kinase, is encircled. The lines represent the confidence levels of the network, stronger associations are represented by thicker lines (STRING database version 10).

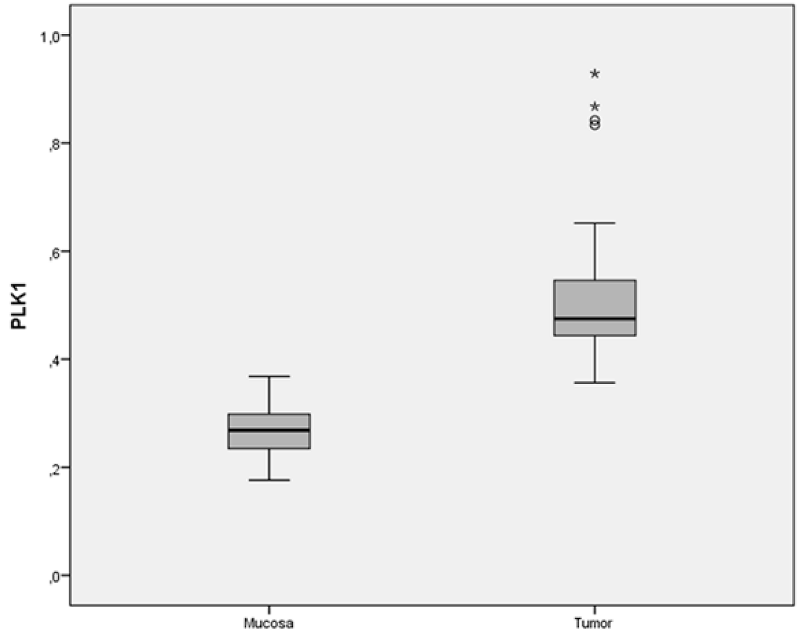

Supplementary Figure 3: Microarray gene expression data revealed elevated levels of *PLK1* in tumor samples. Data derived from 22 pairs of tumor and normal mucosa tissue revealed a 2.26-fold upregulation of *PLK1* expression on average in the tumor samples. Data is presented as boxplots, and the thick lines represent the median values.

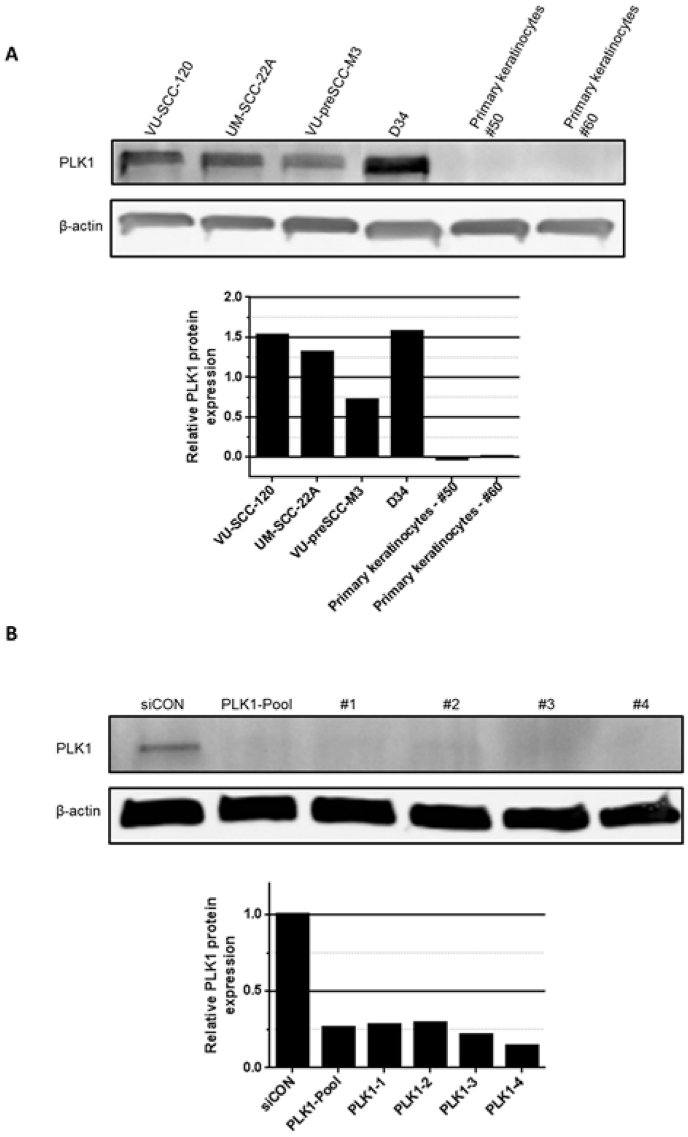

Supplementary Figure 4: PLK1 protein levels in several cell models and after siRNA transfection. (A) Corresponding to the increased *PLK1* expression in tumor material we also found elevated levels of PLK1 protein expression in our (pre)cancer cell models, compared to primary keratinocytes. Bars represent PLK1 expression levels relative to the β-actin levels used as loading control. **(B)** Analysis of PLK1 protein levels in UM-SCC-22A revealed that transfection with the PLK1 SMARTpool and the four individual siRNAs resulted in at least 70% decrease of the protein levels. Bars represent protein expression relatively to the PLK1 protein level of the siCONTROL transfected cells.

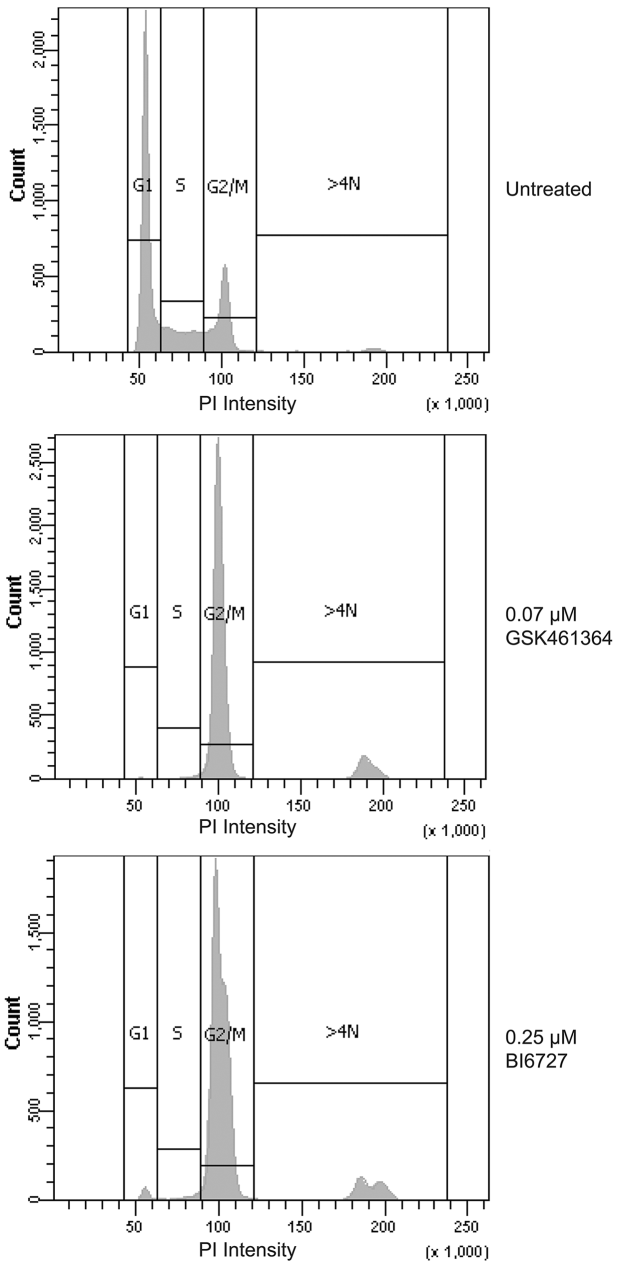

Supplementary Figure 5: Cell cycle profiles of VU-preSCC-M3 show a G2/M arrest upon PLK1 inhibition. 24 hr treatment with either GSK461364 or BI6727 results in a clear accumulation of the preneoplastic cells in the G2/M phase. Shown here are representative figures of one experiment, where PI intensity represents the DNA content.

Supplementary Table 1: Overview of the siRNA re-screen performed on three different cell lines.

See Supplementary File 1

Supplementary Table 2: Characteristics of preneoplastic cell lines and primary cells.

Cell line	Origin	Dysplasia margin/ biopsy	Tumor/ leukoplakia site	TNM (tumor)	Age	Gender	Doubling time cell line (h)
VU-preSCC-M3	Surgical margin	Yes	Glottic larynx	T4aN0	67	M	20
VU-preSCC-1640	Biopsy buccal mucosa	U	Floor of mouth	U	30	M	28
VU-preSCC-HN433	Surgical margin	No	Floor of mouth	T2N2c	66	M	116
VU-preSCC-HN472	Surgical margin	No	Lateral tongue	T1N0	64	M	57
D34	Biopsy leukoplakia	Yes	Lateral tongue	No SCC present	54	F	31
Primary cells	Uvula	No	N/A	N/A	U	U	20-27

Abbreviations: U; unknown, M; male, F; female, SCC; squamous cell carcinoma, N/A; not applicable

Supplementary Table 3: Overview of treatment schedules in the *in vivo* experiment.

Vehicle	Dose	Schedule	Route
Solvent	10% DMSO/10% Solutol	Q2dx6	i.p.
GSK461364	50 mg/kg	Q2dx6	i.p.
BI6727	20 mg/kg	Q7dx3	i.p.
RT/Plat	1x 2 Gray 3 mg/kg Cisplatin	Q7dx2	Total body irradiation i.p.

Abbreviations: i.p.; intraperitoneal
Efficacy of PLK1 small molecule inhibitors was determined in a HNSCC *in vivo* model, according to the indicated schedules. Mice were randomly assigned to one of the groups (n=6, each carrying 2 tumors, in the right and left flank respectively).
